# Dynamic Characterization of Microscopic Pore Structure in Medium–High Permeability Sandstones During Waterflooding

**DOI:** 10.3390/nano15100747

**Published:** 2025-05-15

**Authors:** Jiayi Wu, Wenbo Gong, Qingyu Wang, Yuhang He, Junhui Guo, Yi Bao, Shuai Shao, Rubin Li

**Affiliations:** 1 R&D Center of Sustainable Development of Continental Sandstone Mature Oilfield, Daqing 163712, China; qingyuwang@petrochina.com.cn (Q.W.); hyuhang@petrochina.com.cn (Y.H.); guojunhui@petrochina.com.cn (J.G.); baoyi6@petrochina.com.cn (Y.B.); shaoshuai0857@petrochina.com.cn (S.S.); lirubin8886@petrochina.com.cn (R.L.); 2 Research Institute of Exploration and Development, Petrochina Daqing Oilfield Company, Daqing 163712, China; 3 College of Safety and Ocean Engineering, China University of Petroleum, Beijing 102249, China; 4 Hainan Institute of China University of Petroleum, Beijing 102249, China

**Keywords:** pore classification, local permeable capacity, pore size, pore structure variation, high-PV waterflooding

## Abstract

Understanding the microscopic characteristics and evolutionary patterns of pore structures during high-PV waterflooding is critical for improving the accuracy and efficiency of oil field development. While previous studies have primarily emphasized the geometric and morphological features of overall pore structures, they often overlook local pore-scale properties and their relationship with fluid transport capacity. This study proposes a novel classification method for microscopic pore structures that integrates both pore size and local flow conductivity, enabling a more physically grounded and quantitatively robust evaluation of pore systems across rocks with varying permeabilities. The classification scheme divides microscopic pores into six distinct types based on key parameters such as pore diameter and flow flux area. To validate this approach, high-PV waterflooding experiments were performed on six sandstone samples with different permeabilities. High-resolution micro-computed tomography (micro-CT) imaging was employed to capture the internal pore structures before and after flooding. The results reveal that while low-connectivity small pores dominate numerically across all samples, high-connectivity small pores account for the largest volumetric share in medium-permeability rocks. Although overall pore size distributions remain relatively stable during high-PV waterflooding, transitions between pore types occur, driven by localized structural changes. Notably, in medium-permeability rocks, the number of low-connectivity small pores increases, whereas high-connectivity small pores decline. These findings deepen our understanding of microscopic heterogeneity and provide a theoretical foundation for evaluating the occurrence of residual oil. Moreover, the proposed classification framework offers valuable guidance for optimizing enhanced oil recovery strategies in the late stages of ultra-high water cut development.

## 1. Introduction

Most of the medium-high permeability sandstone oilfields in China have entered the ultra-high water cut stage where challenges such as rising water production, reduced oil recovery efficiency, and difficulties in exploiting residual oil have become increasingly prominent. In this context, developing advanced strategies for residual oil recovery and improving the overall recovery factor has become a critical research focus [1,2,3]. At this stage, residual oil is typically characterized by spatially dispersed and locally concentrated distributions, shaped by the complex interplay between oil and water during multiphase flow in the reservoir [4,5]. The storage and transport capacities of reservoir rocks are fundamentally governed by their microscopic pore structures [6,7,8,9,10], which serve not only as reservoirs for fluids but also as primary flow conduits. Heterogeneity in pore structure significantly influences fluid distribution, migration, and ultimately the amount of residual oil. Therefore, accurate pore structure characterization is essential for designing enhanced oil recovery (EOR) strategies and addressing engineering problems such as targeted residual oil exploitation and subsurface fluid treatment.

Medium-to-high-permeability sandstones typically exhibit complex and irregular microscopic pore geometries, making their quantitative description a longstanding challenge [11,12]. Pore structure characterization commonly includes assessments of pore size, shape, spatial distribution, and interconnectivity. A range of imaging and experimental techniques have been developed for this purpose [13]. Imaging-based approaches—such as cast thin sections, scanning electron microscopy (SEM), X-ray computed tomography (CT), focused ion beam SEM (FIB-SEM), and synchrotron radiation—enable direct visualization and quantitative analysis of pore geometries and heterogeneities [14,15,16,17,18]. Experimental methods—such as high-pressure mercury intrusion, gas adsorption, nuclear magnetic resonance (NMR), and small-angle scattering—provide parameters that indirectly reflect the internal pore structure [19,20,21,22]. Despite the progress of these methods, most focus primarily on static geometric attributes, with limited capability to assess the actual contribution of pores to fluid flow. This limitation hinders the connection between pore-scale analysis and macroscopic recovery performance. To better understand how pore structures influence residual oil retention and mobilization, it is critical to evaluate them from a fluid flow perspective.

Recent studies have examined the morphological characteristics and size distributions of pores in sandstone reservoirs [23,24,25,26], contributing valuable insights through porosity and permeability measurements, as well as pore structure metrics such as average radius, connectivity, heterogeneity index, and anisotropy. For instance, Ren et al. [27] investigated pore-throat distributions and their influence on permeability in sandstone reservoirs of varying quality using casting thin sections and capillary pressure curves, emphasizing the role of submicron features. However, they did not explore how different pore types within the same core contribute differently to flow. Similarly, Guo et al. [28] analyzed dominant pore structures and throat controls on fluid mobility across reservoirs with different permeability levels using conventional core analysis, NMR, and gas–water centrifugation, but lacked detail on the variability of mobility across pore types. Pei et al. [29] compared core data obtained from cast sections, SEM imaging, and mercury intrusion to identify differences in micropore structures, yet did not elaborate on differences in permeation capacity among pore types.

Collectively, these studies affirm the importance of quantitatively evaluating pore structures to better understand residual oil occurrence in sandstone reservoirs. However, a clear link between microscopic pore characteristics and fluid transport behavior remains lacking, particularly in the context of long-term waterflooding. Moreover, understanding how pore structures evolve under high-pore-volume (high-PV) waterflooding is further complicated by the unclear relationship between structural features and dynamic flow behavior.

To address this knowledge gap, this study proposes a novel classification method for microscopic pore structures that incorporates both geometric parameters (e.g., pore size) and dynamic characteristics (e.g., flow flux area). By considering local flow capacity, this dual-parameter classification allows for a more accurate distinction between productive and non-productive pore networks. A series of high-PV waterflooding experiments were conducted on six natural sandstone cores, followed by high-resolution CT imaging and digital rock reconstruction. The pore structures were analyzed and classified based on the proposed method. This work also investigates the temporal evolution of pore structures under waterflooding, focusing on the impact of structural heterogeneity while controlling for geochemical or mineralogical effects such as clay swelling or dissolution–precipitation reactions. Although these effects are acknowledged as significant, they are beyond the scope of this image-based structural analysis.

## 2. Materials and Methods

### 2.1. Sandstone Rock Samples

According to the Evaluation Methods for Oil and Gas Reservoirs (SY/T 6285-2011), which is an oil and gas industry standard of the People’s Republic of China, medium-to-high-permeability sandstone reservoirs are classified into three categories: (1) ultra-high-permeability reservoirs with air permeability equal to or greater than 2000 mD; (2) high-permeability reservoirs with air permeability ranging from 500 to 2000 mD; and (3) medium-permeability reservoirs with air permeability between 50 and 500 mD. Based on the permeability, porosity, and median particle size of reservoir rocks in the Songliao Basin, two representative core samples from each category—ultra-high-, high-, and medium-permeability—were selected for analysis. The physical properties of these sandstone samples were measured experimentally, and the results are summarized in Table 1.

### 2.2. Analysis in the Representative Element Volume of Porous Structures

**Figure A1:**
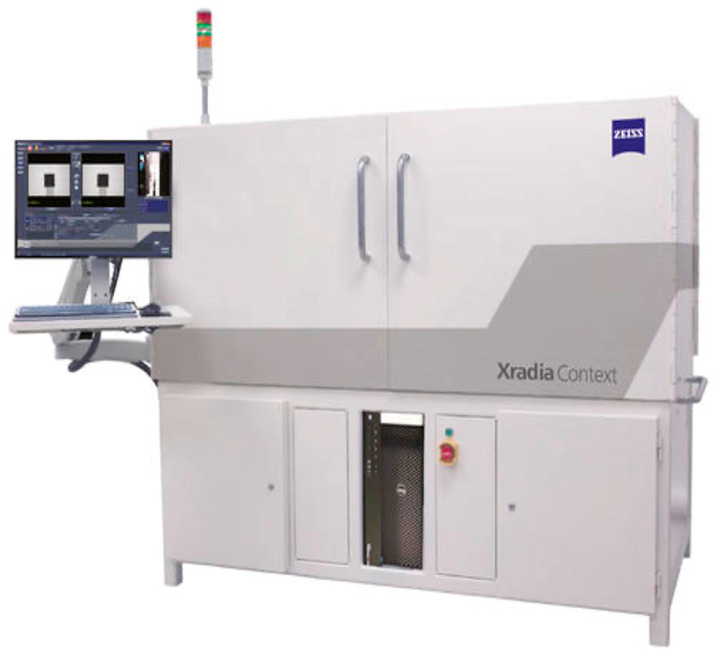
The experimental apparatus for micro-CT (X-radia MicroXCT-400).

This study combines micro-CT scanning with waterflooding oil displacement experiments to non-destructively analyze core samples at different stages of the displacement process, and the experimental setup is displayed in Figure A1 in Appendix A. Micro-CT imaging at multiple stages enables detailed visualization of pore structure evolution across samples with varying permeability. Digital rock models were reconstructed from the CT images, allowing for pore-scale analysis of structural characteristics and their changes during displacement.

Two key trade-offs must be considered prior to CT scanning. First, there is an inverse relationship between sample size and scanning resolution: increasing the sample size leads to a reduction in resolution, potentially obscuring fine structural details, whereas pursuing higher resolution limits the scan size, which may result in inadequate coverage of representative elementary volume (REV) and compromise the representativeness of the structure. Second, scanning cost increases with resolution. Given that smaller pore channels contribute minimally to fluid flow under practical waterflooding conditions, achieving a resolution beyond a certain threshold provides limited added value. Therefore, from an engineering standpoint, it is sufficient to meet a practical resolution threshold without unnecessarily increasing scanning cost.

**Figure 1:**
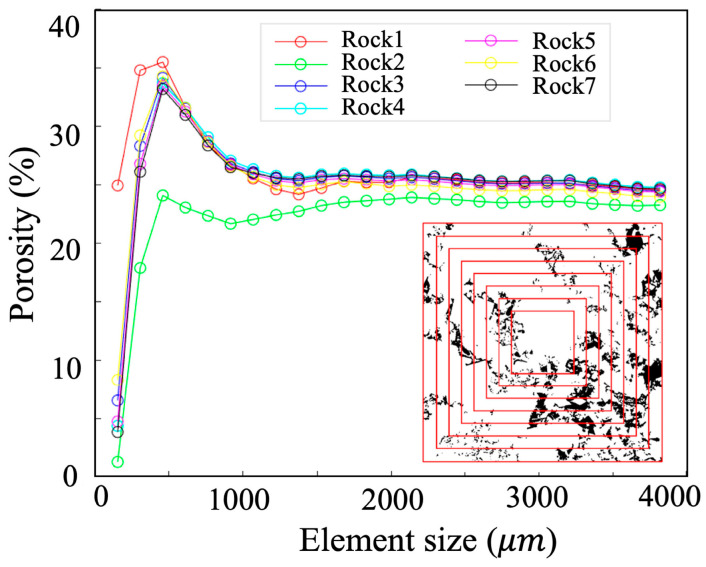
Changes in porosity of digital rocks extracted from the same rock sample at different sizes. The element size crop is labelled by the red line in the subfigure and the colored markers represent different rocks from published data [26].

To accurately characterize pore structures using digital rock models, this study aimed to determine the minimum REV and corresponding resolution threshold for sandstone reservoirs with different permeability levels. Permeability was chosen over porosity as the evaluation criterion in REV analysis, as it directly reflects the fluid flow capability of the porous media, while porosity merely describes the volumetric fraction of pore space. Determining the REV provides insights into the reliability and representativeness of digital rock models derived from micro-CT scans. To identify the REV, fluid flow simulations were conducted on subdomains of increasing size, extending from the core center outward. The average permeability within each subdomain was computed to evaluate the convergence behavior. As illustrated in Figure 1, when the domain size is smaller than the REV, the computed permeability fluctuates significantly due to insufficient statistical pore representation. Once the domain reaches the REV, the average permeability stabilizes and reflects the bulk behavior of the porous medium.

**Figure 2:**
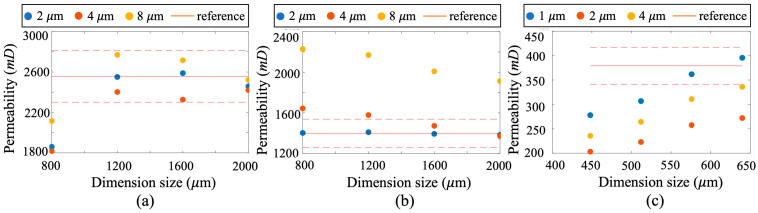
Simulated results of rock samples at different resolution sizes and domain sizes for the sandstone samples with varied permeabilities: (**a**) ultra-high permeability sample; (**b**) high-permeability sample; and (**c**) medium-permeability sample. The red line labelled as reference represents the experimental measurements.

The lattice Boltzmann method (LBM) was employed to simulate fluid flow within the reconstructed porous structures [30,31,32]. Simulation parameters are listed in Table 2. Through these simulations, the effect of domain size and scanning resolution on the calculated permeability was analyzed, enabling evaluation of the REV and suitable scanning conditions. To investigate the influence of resolution, core samples from each permeability class were scanned at resolutions ranging from 1 µm to 8 µm per voxel. For each resolution level, multiple subdomains of varying size, centered at the same location, were extracted. LBM simulations were conducted in these subdomains to obtain the gas velocity field, from which permeability was calculated using Darcy’s law. The simulation results are presented in Figure 2: (1) panels (a), (b), and (c) correspond to the permeability results for ultra-high-, high-, and medium-permeability samples, respectively; (2) blue, red, and yellow symbols represent results at scanning resolutions of 2, 4, and 8 µm/voxel, respectively; and (3) due to the smaller pore size of the medium-permeability samples, additional scans were performed at 1 µm/voxel.

The red solid lines in Figure 2 indicate experimentally measured permeability values (from Table 1), while the red dashed lines show the 10% deviation range used as the acceptable error threshold in engineering practice. Comparison between simulation and experimental results indicate that as the dimension size increases and scanning resolution size decreases, the calculated permeability stabilizes, and the difference between the calculated and measured values decreases. This proves that a digital rock model constructed at a reasonable size can effectively determine fluid flow characteristics within the interior pore structure of the rock core. Considering both cost and acceptable accuracy in engineering applications, maintaining the calculated permeability within a 10% deviation of the experimental value is considered sufficient for determining the REV and critical scanning resolution. Notably, REV and resolution requirements for different reservoir types could be specified as follows: (1) for ultra-high-permeability reservoirs, the minimum REV should be no less than 1200 µm, and the resolution should be finer than 8 µm/voxel; (2) for high-permeability reservoirs, a minimum REV of 800 µm and a resolution finer than 4 µm/voxel are required; and (3) for medium-permeability reservoirs, the minimum REV should be at least 600 µm, with a resolution of no more than 2 µm/voxel to capture the finer pore structures accurately. It is important to note that while REV analysis ensures representativeness at the pore scale, it only reflects local heterogeneity and does not fully capture large-scale geological variability.

## 3. Results and Discussion

### 3.1. Pore Size Distribution of Porous Structures

**Figure 3:**
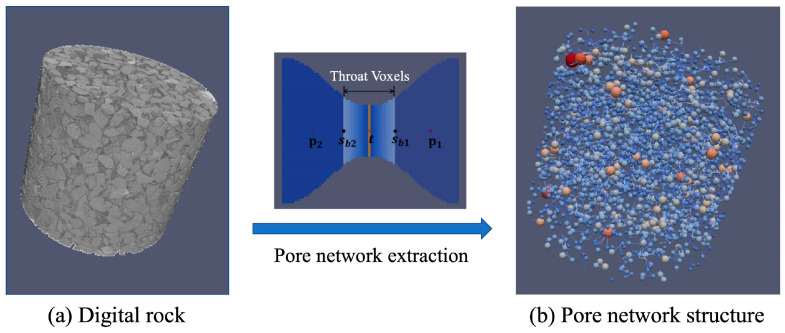
Pore network extraction from CT-scanning digital rock to a 3D-network structure.

To further investigate the connectivity of the microporous structures and the characteristics of fluid flow within them, a pore network model was constructed using a pore structure segmentation method, as illustrated Figure 3. The left panel of the figure shows the grey images of rock sample. In this image, the gray regions represent the rock matrix composed of various minerals, the black regions within the matrix correspond to the pore spaces, and the black area outside the matrix denotes the image background. The segmentation method simplifies the complex porous structure into a network of pore bodies (modeled as spheres) and pore throats (modeled as cylinders), based on the local pore-throat geometry. This approach, shown in the right panel of Figure 3, is rooted in the foundational theory proposed by Fatt [33] and has undergone significant refinement over the past two decades, becoming a widely adopted technique for constructing pore network models.

**Figure 4:**
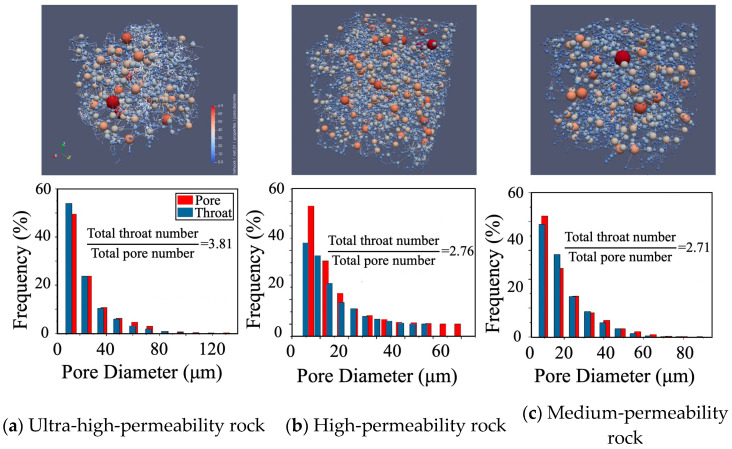
Extraction of core-pore network model and pore-throat distribution in the reservoir rocks with different permeabilities.

**Figure 5:**
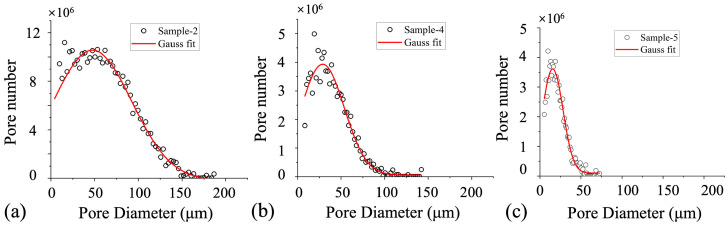
Pore diameter distribution in the reservoir rocks with different permeabilities: (**a**) Sample 2; (**b**) Sample 4; (**c**) Sample 5.

In the pore network diagrams, pore bodies are represented as spheres, with their size and color indicating the pore radius. The lower panels of Figure 4 present the percentage distributions of pore bodies and pore throats for each sample, shown as red and blue bars, respectively. Characteristic parameters of the pore structures, extracted from these models and illustrated in Figure 5, reveal that the ultra-high-permeability reservoir features a wide range of pore sizes—from 10 µm to 130 µm in diameter—with a peak distribution around 50 µm. This sample also exhibits high pore-to-throat connectivity, with a total throat-to-pore ratio of 3.81. It is important to note that the pore size distributions shown in Figure 5 represent the combined size distribution of pore bodies and throats in Samples 2, 4, and 5, which correspond to the ultra-high-, high-, and medium-permeability reservoir rocks, respectively. As permeability decreases, high-permeability sandstones show a narrower pore size distribution (10 µm to 80 µm in diameter, with a peak around 32 µm) and more refined pore throats, resulting in a lower throat-to-pore ratio of approximately 2.76. Medium-permeability reservoirs are characterized by a greater abundance of smaller pores and throats, with pore sizes clustered around 18 µm, and a further reduction in the throat-to-pore ratio to approximately 2.71. This decrease in throat dimensions generally leads to reduced permeability.

### 3.2. A New Classification of Local Pore Structure

The physical characteristics of pore structures, particularly the permeable capacity, plays a significant role in controlling fluid flow paths at both local and global scales in sandstone rocks. These properties directly impact the permeability of rocks and, consequently, influence the migration behavior of subsurface fluids such as oil and water. Previous pore classification methods [34,35,36,37] have primarily focused on morphological and geometrical parameters such as average throat radius, sorting coefficient, and homogeneity coefficient. Although these descriptions offer detailed insights into the geometric features of porous structures, they often overlook the direct influence of local pore connectivity on flow capacity. However, in the context of subsurface oil and gas recovery, it is critical to characterize the porous structure from the perspective of fluid flow, especially regarding the contribution of localized pore configurations to overall rock permeability. A more nuanced understanding of such structures can provide meaningful guidance for optimizing enhanced oil recovery strategies.

**Figure 6:**
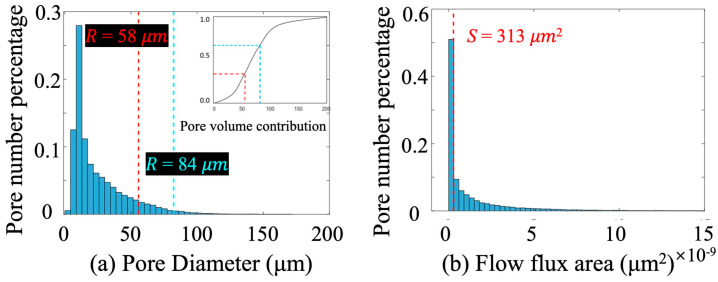
Thresholds of pore structure classification parameters: (**a**) pore diameter, where the critical value is specified by the pore volume contribution with 1/3 and 2/3, as labelled by red and green dash lines; (**b**) flow flux area.

To better understand the functional impact of pore structure on fluid flow, we introduce a classification framework that combines morphological and dynamic descriptors. Specifically, pore size reflects the local storage and intrinsic permeability potential, while the local flow flux area accounts for the cumulative cross-sectional area of connecting throats, serving as a proxy for flow transmission capacity. This composite characterization allows us to not only differentiate pores based on their geometry but also to evaluate their role in facilitating or restricting fluid flow across the pore network. As such, the method bridges the gap between structural descriptors and flow-relevant performance metrics, offering a more comprehensive view of microstructural control on macroscopic transport phenomena. The threshold for these two key descriptors can be easily identified through statistical analysis of pore network characteristics in digital rock models, as illustrated in Figure 6.

Pore diameter R: This parameter is used to represent the size distribution and geometrical morphology of granular pores in the sandstone. Pore diameter directly affects the hydraulic resistance experienced by fluids during interior fluid flow in a pore structure. To classify the pore system, we constructed a pore diameter–volume percentage curve based on the analysis of throat diameter distributions in sandstone samples with varying permeability levels. The pore system was divided into three categories—large, medium, and small pores—according to the volume-weighted distribution. Specially, pore diameter corresponding to 33.3% and 66.6% cumulative pore volume were selected as boundaries. As shown in Figure 6, diameters of 58 μm and 84 μm served as critical thresholds. Pores with diameter smaller than 58 μm were classified as small pores, ones larger than 84 μm as large pores, and those in between as medium pores.

Flow flux area S: To evaluate the transport capacity of local pore structures, it is essential to introduce the effect of connected pore throats. The cross-section area of these throats serves as a proxy for local flow capacity. For a pore body connected to *N* throats, we define a new parameter, the flow area $A_{t}$, as the summary of the cross-section area $A_{i}$ of all connected throats:

$$A_{t}=\sum_{i=1}^{N}A_{i}$$

This parameter captures both the degree of connectivity and the transport potential of each pore, making it a more direct indicator of local permeability. It also aids in understanding the role of pore-throat structures in influencing heterogeneous fluid migration. Based on the distribution shown in Figure 6, the median flow flux area from this curve (313 μm^2^) was selected as the threshold to distinguish between high-connectivity and low-connectivity pores.

Utilizing the defined thresholds of pore diameter and flow flux area, we propose a comprehensive classification scheme for micropore structures in medium-high permeability sandstone. Pores are subdivided into six types: high-connectivity large pores, low-connectivity large pores, high-connectivity medium pores, low-connectivity medium pores, high-connectivity small pores, and low-connectivity small pores, as shown in Table 3. This classification framework enables an effective categorization of pore structures across sandstone samples with different permeability levels. It serves as a basis for evaluating the evolution of pore structure and the associated fluid flow behavior during high-PV waterflooding process.

It should be noted that the pore structure classification model developed in this study is based on several key assumptions:

(1) Representative Sampling: It is assumed that for each permeability range within the target sandstone reservoir, a representative core exists whose pore structure reflects typical flow characteristics for that reservoir zone. In this study, one core was selected from both medium-permeability and high-permeability zones.
(2) Representative Elementary Volume (REV): It is assumed that an REV can be established for each selected core, allowing for stable statistical description of microstructural features within a finite digital volume.
(3) Low-Velocity Flow Regime: The model assumes low flow velocities, under which the effects of inertial forces and non-laminar behavior can be neglected. This assumption is consistent with flow conditions in subsurface reservoirs.
(4) Image Resolution Constraints: The digital core reconstructions are based on high-resolution CT scans, which inherently limit the smallest resolvable features. Therefore, pores and throats below the resolution threshold (typically <1 µm) are not included in the analysis. This implies that the model may underestimate total porosity or misrepresent nanoporous domains in real rock.
(5) Flow-Relevant Parameters: The classification relies on pore diameter and flow flux area as key indicators of local flow capacity and connectivity. It is assumed that these geometric features adequately capture the relevant flow heterogeneity at the pore scale.

**Figure 7:**
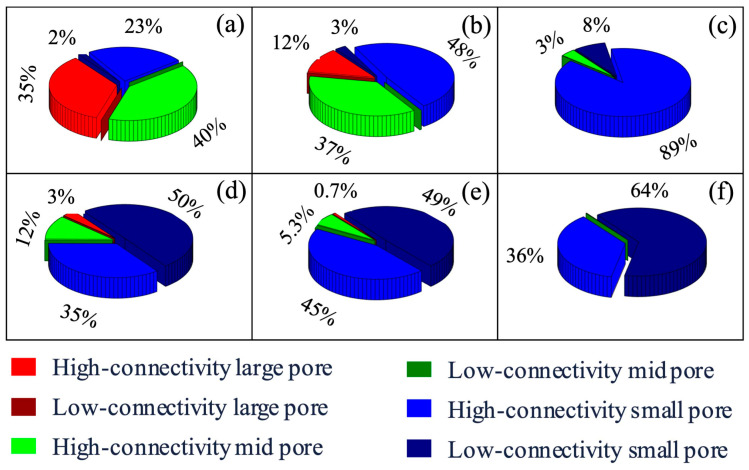
Proportion of different types of pores in the reservoirs rocks with various permeabilities: (**a**–**c**) for volume proportion of pores in Sample 2, Sample 4, and Sample 5, respectively; (**d**–**f**) for number proportion of pores in Sample 2, Sample 4, and Sample 5, respectively.

These assumptions define the scope and applicability of the proposed classification model and are particularly relevant for digital rock analysis of moderately to highly permeable sandstone reservoirs. Based on the aforementioned pore structure classification method, this study categorized and comparatively analyzed the pore structures of ultra-high-, high-, and medium-permeability reservoirs, identifying the distinct distributions of pore types across reservoirs with varying permeabilities, as illustrated in Figure 7. During the imaging process, pore characteristics such as diameter and flow area were quantified. Each pore was then classified according to the micropore structure criteria outlined in Table 4. Specially, the pores of high-connectivity large pores, low-connectivity large pores, high-connectivity medium pores, low-connectivity medium pores, high-connectivity small pores, and low-connectivity small pores are labelled as 1, 2, 3, 4, 5, and 6, respectively, and other solid parts are labelled as 0. Using digital rock analysis, the volume and number percentages of each pore type were determined by quantifying the occurrence of labels 1 through 6 within the segmented digital rock images of reservoir samples with different permeabilities.

From a volumetric perspective, ultra-high- and high-permeability reservoir rocks are dominated by high-connectivity medium-to-large pores, indicating favorable storage and flow capacities. In contrast, medium-permeability reservoirs are primarily composed of small pores, suggesting that effective connectivity within small pore networks is critical to overall permeability. In terms of pore number distribution, low-connectivity small pores are prevalent across all reservoir types. However, their impact on fluid flow varies significantly with permeability. In ultra-high-permeability rocks, over 50% of the pores are classified as low-connectivity small pores, yet they contribute only about 2% to the total pore volume, thus having negligible influence on oil–water flow dynamics. Conversely, in medium-permeability reservoirs, low-connectivity small pores comprise approximately 64% of the total pore count and contribute up to 10% of the pore volume, thereby playing a more significant role in impeding fluid movement. After excluding non-connected pores, the connected pore networks in all reservoir samples exhibited good continuity; however, the distribution of pore sizes varied markedly. In ultra-high-permeability reservoirs, high-connectivity large pores dominate, accounting for approximately 50% of the total pore volume. In contrast, in medium-permeability reservoirs, the volume fraction of high- and medium-connectivity large pores drops sharply to less than 1%, with only a minimal number of such pores present. This indicates a structural transition in medium-permeability reservoirs, where high-connectivity small pores become dominant, ultimately reducing the overall seepage capacity of the rock.

### 3.3. Porous Structure Variation During Long-Term Waterflooding

Following the determination of the minimum representative elementary volume (REV) and optimal scanning resolution for sandstone samples with varying permeabilities, high-pore-volume (high-PV) waterflooding experiments were conducted. Imaging was performed using an X-radia MicroXCT-400 X-ray microscope equipped with a high-resolution detector (2048 × 2048 pixels) and a spatial resolution of 1.91 μm. The vertical field of view encompassed 2000 image slices. The experimental procedure for the high-PV waterflooding tests is summarized in Table 4.

To enhance grayscale contrast and facilitate accurate phase segmentation in the CT images, a brine solution containing 10 wt% sodium iodide was used as the water phase, while liquid paraffin was selected as the oil phase due to the significant difference in their X-ray attenuation properties. The water phase had a density of 1.12 g/cm^3^ and a viscosity of 1.2 mPa·s, while the oil phase had a density of 0.84 g/cm^3^ and a viscosity of 7.5 mPa·s. The interfacial tension between the oil and brine phases was 30 mN/m. The wettability of the sandstone samples was characterized by measuring the average contact angle at the three-phase contact line in samples saturated with oil and brine. The resulting contact angles for Samples 2, 4, and 5 were 83.1°, 80.8°, and 76.8°, respectively, indicating a weakly hydrophilic condition. During sample preparation, the dried sandstone cores were vacuumed and saturated with brine, then subjected to a pressurization of 20 MPa for 7 days. Each core was subsequently placed in a core holder connected to the immiscible displacement experimental system. To simulate oil–water coexistence within the pore structure, the oil phase was injected into the brine-saturated rock at a constant flow rate of 11 μm/s, under ambient temperature (25 °C) and a confining pressure of 5 MPa, until water was no longer produced at the outlet. The system was then allowed to equilibrate for 12 h. The initial fluid distribution was recorded at 0 PV (pore volume, defined as the ratio of the injected fluid volume to the pore volume of the rock). The waterflooding experiment involved brine injection into the core sample, with CT imaging performed at seven displacement stages: 0 PV, 0.5 PV, 1 PV, 3 PV, 10 PV, 50 PV, and 500 PV. The injection was conducted in three sequential phases with increasing flow rates: from 0 PV to 3 PV at 11 μm/s, from 3 PV to 10 PV at 33 μm/s, and from 10 PV to 500 PV at 110 μm/s. During post-processing, CT scan data from each displacement stage were segmented to isolate and extract the oil, water, and solid phases using specialized image analysis software, ImageJ (https://imagej.net). This enabled the dynamic quantification of fluid distributions and structural variations within the digital rock models at the pore scale.

Following the determination of the minimum REV and optimal scanning resolution size for sandstone rocks with different permeabilities, this study proceeded to perform long-term waterflooding experiments. The rock and fluids were imaged using an X-radia MicroXCT-400 X-ray Microscope, equipped with a high-resolution microscopy detector featuring a pixel size of 2048 × 2048 and a resolution of 1.91 μm. The vertical view field comprises 2000 image slices. Table 4 outlines the experimental process for long-term waterflooding in sandstone rocks. To enhance grey value contrast and facilitate phase segmentation in CT images, brine containing 10 wt % sodium iodide was used as the water phase, while liquid paraffin was selected as the oil phase due to their contrasting X-ray intensities. The fluid densities and viscosities were 1.12 g/cm^3^ and 1.2 mPa·s for the water phase, and 0.84 g/cm^3^ and 7.5 mPa·s for the oil phase, respectively. The oil–brine interfacial tension was 30 mN/m. The wettability of the sandstone samples was determined by measuring the average contact angle from the three-phase contact line in rock samples saturated with oil and brine, resulting in contact angles of 83.1°, 80.8°, and 76.8° for Samples 2, 4, and 5, respectively, indicating a weakly hydrophilic state. During experimental preparation, the dried sandstone core was vacuumed, saturated with brine, pressurized to 20 MPa for 7 days, and then placed into a core chamber connected to the immiscible displacement experimental system. To simulate water–oil coexistence in the rock, the oil phase was injected into the brine-saturated rock sample at a flow rate of 11 μm/s, a temperature of 25 °C, and a confining pressure of 5 MPa until no water was produced at the outlet. After allowing the system to equilibrate for 12 h, the initial water–oil distribution was recorded at 0 PV (i.e., pore volume, the ratio of injected fluid volume to the volume of pore structure). The long-term waterflooding experiment involved brine injection into the rock sample, with pore structure and water–oil distribution information captured at seven displacement stages using CT scanning. The stages were 0 PV, 0.5 PV, 1 PV, 3 PV, 10 PV, 50 PV, and 500 PV. Three sequential injection phases with different velocities were performed: 0 PV to 3 PV at 11 μm/s, 3 PV to 10 PV at 33 μm/s, and 10 PV to 500 PV at 110 μm/s. For the imaging process, the scanning data information at different displacement stages were segmented to isolate and extract the oil, water, and solid rock phases using the specialized image analysis software such as Avizo (Version 2024.2) and ImageJ. This process achieved the dynamic quantification of the microporous structural variations in the digital rock models.

#### 3.3.1. Statistical Characteristics Variation of Porous Structure

**Figure 8:**
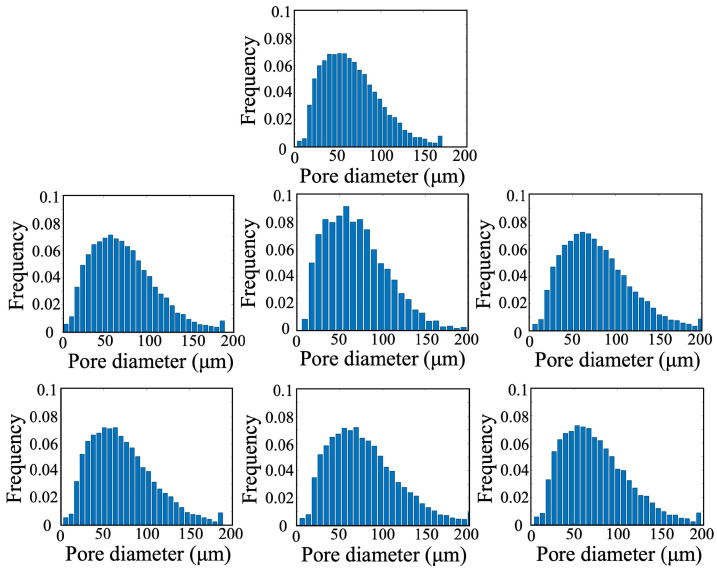
Pore size distributions of porous structure at different displacement stages (0 PV, 0.5 PV, 1 PV, 3 PV, 10 PV, 50 PV, and 500 PV) during long-term waterflooding (taking Sample 1 as an example).

During the high-PV waterflooding experiments, the previous studies mainly focus on the water–oil distribution in porous structure of the rocks. In fact, due to the impact of clay expansion with water adsorption and the erosion detachment of fine particles, the porous structure usually changes during the waterflooding. To investigate the evolution of pore structures in sandstone samples with varying permeabilities under prolonged waterflooding, this study analyzed the pore size distributions derived from digital rock models at multiple displacement stages (0 PV, 0.5 PV, 1 PV, 3 PV, 10 PV, 50 PV, and 500 PV), as illustrated in Figure 8.

**Figure 9:**
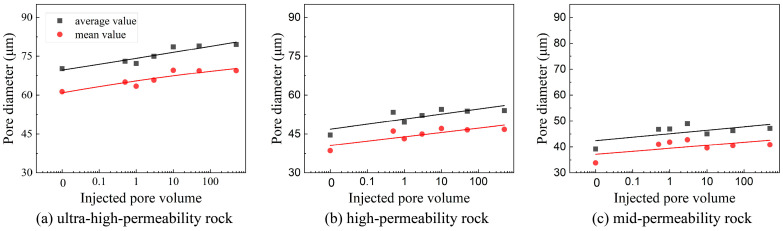
Varying characteristic of pore size in the digital rocks with different permeability during long-term waterflooding: (**a**) Sample 2; (**b**) Sample 4; and (**c**) Sample 5.

**Figure 10:**
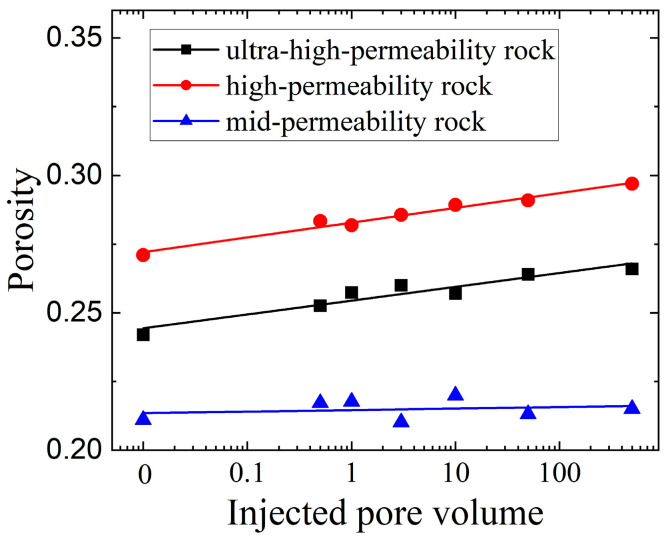
Porosity variations in reservoir cores under long-term waterflooding. The horizontal axis means the ratio of injected water volume to the whole volume of porous system.

Across all core samples, the pore size distributions exhibited similar trends, spanning a wide range from 0 μm to 200 μm. Notably, at the 500 PV stage, the distribution of pore sizes in the 130–160 μm range showed a moderate increase. As displacement progressed, both the mean and median pore diameters increased consistently for all sandstone types. The magnitude of these increases was more pronounced in ultra-high- and high-permeability samples, while medium-permeability rocks exhibited minimal variation in pore diameter, as shown in Figure 9. A strong linear correlation was identified between porosity and the cumulative pore volume of injected fluid (PV) at various stages. In ultra-high- and high-permeability sandstones, porosity generally increased with continued water injection, indicating enhanced pore connectivity and improved permeability during high-PV waterflooding. In contrast, porosity in the medium-permeability sandstone remained largely unchanged throughout the experiment, as presented in Figure 10.

#### 3.3.2. Evolution Characteristics for Different Pore Types

**Figure 11:**
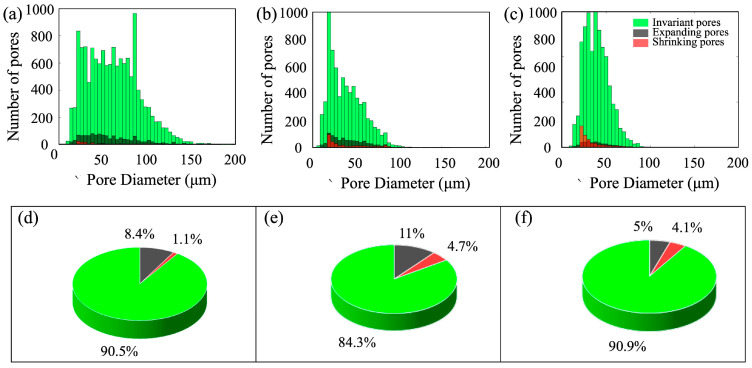
Evolution of porous structure during long-term flooding, (**a**–**c**) for the number of pores with size variation for ultra-high-permeability, high-permeability and medium-permeability rocks, respectively, and (**d**–**f**) for the corresponding quantity percentage of pores with size variation.

This section characterizes the complex variations in reservoir rock pore structures by examining local changes in pore size throughout the displacement process. To capture the dynamic evolution of various pore types, we extracted and analyzed the volume and number proportions of different digital rock models with varying permeabilities at key displacement stages—0 PV, 0.5 PV, 1 PV, 3 PV, 10 PV, 50 PV, and 500 PV—as illustrated in Figure 11. Pore size variation was determined by comparing pore diameters at each displacement stage with their initial values. Based on these comparisons, pores were categorized into three groups: enlarged pores, unchanged pores, and reduced-size pores. Considering the resolution and inherent uncertainties of digital rock imaging, pores with size changes below 1% were classified as invariant.

As shown in Figure 11, pore expansion was more prevalent in ultra-high-permeability rocks, while medium-permeability rocks exhibited a higher proportion of pore contraction, likely due to clay swelling and particle plugging. Although over 84% of pores remained unchanged across all samples, limited but notable local changes were observed after high-PV waterflooding, which are attributed to mechanical effects such as particle migration or clay expansion [38,39,40], rather than chemical dissolution. While this study emphasizes pore classification from a flow heterogeneity perspective, we recognize that other mechanisms—such as clay swelling, mineral dissolution, and fluid–rock chemical reactions during prolonged water injection—can significantly influence pore-scale characteristics. Preliminary core-scale analyses at different water-cut stages revealed changes in wettability and mineral composition, highlighting the role of fluid–rock interactions in microstructural evolution. However, incorporating these processes into a fully quantitative modelling framework would require dedicated experiments and coupled reactive transport simulations, which are beyond the scope of the present work. Future studies will aim to integrate these mechanisms to further refine the proposed classification methodology.

**Figure 12:**
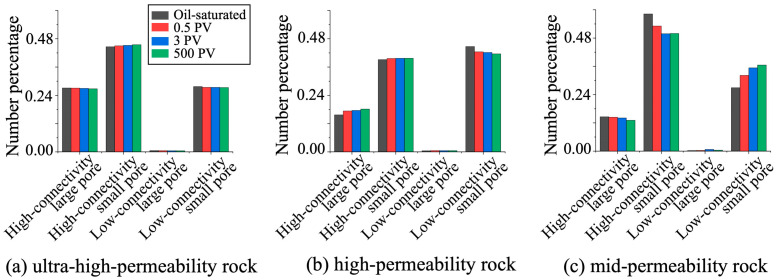
Changes in pore size distribution and proportions of classified pores in different reservoirs under long-term waterflooding.

**Figure 13:**
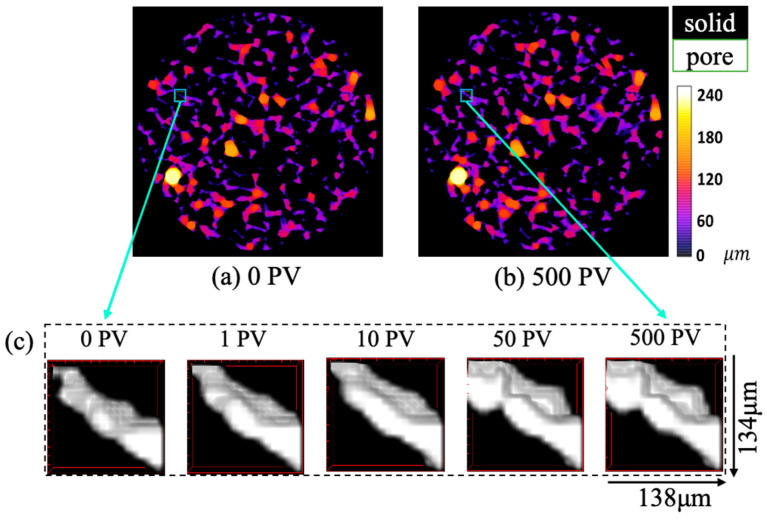
The local variation of pore structure in Sample 2 with ultra-high permeability: a planar view of the diameter of each pore at (**a**) the initial stage (0 PV), and (**b**) the final stage (500 PV), where the colored variation represents the pore diameter distribution in the local pore structure. Subfigure (**c**) shows a 3D view of the local pore structure with domain size 134 × 138 μm from 0 PV to 500 PV, where the black part stands for the solid matrix and the white part for the pore space.

To deepen our understanding of pore size evolution under waterflooding, the variations across different pore types are further presented in Figure 12. This figure includes both the global pore size distribution and the local distribution of labelled pore types (0–6) at different displacement stages. Our findings show that micro-heterogeneity increases in all sandstone samples as the displacement progresses. In particular, significant expansion occurs in medium- and large-sized pores, especially in rocks with ultra-high and high permeability. In these samples, pores larger than 25 μm exhibit prominent growth, which enhances the connectivity of high-permeability pathways and extends the spatial reach of dominant flow channels, thereby intensifying preferential flow. Local pore structure changes are visualized at various stages in Figure 13. In ultra-high-permeability rocks, two-dimensional slices reveal minimal differences in overall pore diameter distribution between the initial (0 PV) and final (500 PV) stages. However, localized enlargement of narrow pore channels is evident, which aligns with prior studies attributing such changes to mineral migration [38]. In contrast, medium-permeability rocks show pore size variations mainly within the 20–40 μm range. Although overall pore connectivity improves, the increased abundance and distribution of low-connectivity small pores suggest a more intricate connectivity network. This enhanced interconnection among small pores contributes to complex fluid transport dynamics, though the connectivity benefits from large pores are not as significant as in higher-permeability rocks. The distinct pore evolution pattern in medium-permeability reservoirs reflects a unique structural response to waterflooding.

These insights provide a scientific basis for understanding the distribution of microscopic residual oil and formulating optimized strategies for enhanced oil recovery. They also improve our understanding of pore structure evolution across different reservoir types during displacement processes. It should be noted, however, that this study is based on core-scale samples with limited spatial coverage. While the results offer valuable pore-scale insights and representative elementary volume (REV) estimates across permeability levels, they may not fully capture the structural heterogeneity at the reservoir scale. Future research involving larger-scale data sets and statistical upscaling techniques will be essential to bridge this gap.

## 4. Conclusions

In this study, the complex pore structures of sandstone reservoir rocks were systematically characterized using a novel microscopic classification method that employs pore diameter and flux area as dual key parameters. This approach captures the complexity of porous structure from both a global perspective (pore size distribution, averaged pore size, and porosity) and a local perspective (pore diameter, flow flux area, and pore type). Additionally, this method illustrates the evolution of pore structures during high-PV waterflooding in reservoir rocks with different permeabilities.

Sandstone rocks with permeabilities ranging from 200 mD to 4000 mD exhibit significant differences in pore size distribution and throat connectivity. With decreasing permeability, rocks exhibit more numerous small pores and finer throats, leading to increased flow resistance and reduced effective permeability. To describe such variability, the study introduced the flow flux area as an indicator of local throat transport capacity, and established a six-type classification scheme: high-connectivity large pores, low-connectivity large pores, high-connectivity medium pores, low-connectivity medium pores, high-connectivity small pores, and low-connectivity small pores. This scheme quantitatively reflects variations in local flow capacity and network connectivity.

The results show that high-permeability rocks are dominated by large pores with medium-to-high connectivity, yielding superior storage and flow capabilities. In contrast, low-permeability rocks contain more small, poorly connected pores, resulting in weaker permeability and enhanced microstructural heterogeneity. High-PV waterflooding improves the connectivity of dominant pores in high-permeability rocks, while in low-permeability rocks, it leads to the proliferation of low-connectivity small pores, increasing flow complexity despite global connectivity gains.

Although this study focuses on medium-to-high-permeability sandstone reservoirs, the proposed classification method—centered on flow heterogeneity—holds promise for adaptation to other reservoir types, including tight sandstones, carbonates, and fractured systems, where pore-scale dynamics and nonlinear flow behaviors differ markedly. By incorporating representative digital rock data and recalibrating classification parameters, the framework can be extended to capture flow-relevant features across diverse geological settings. Moreover, coupling this method with digital rock reconstruction, multiscale simulations, and geological modelling may help establish quantitative relationships between pore structure and reservoir-scale flow behavior. However, the current approach is based on core-scale analysis and is not directly applicable to field-scale simulations. Nonetheless, it provides a foundational methodology and valuable pore-scale insights that can support upscaling efforts and inform more accurate field-scale predictions.

## Figures and Tables

**Table 1:** Physical properties of rock samples.

Sample Number	Reservoir Type	Lithologic Feature	Porosity (%)	Permeability (10^−3^ μm^2^)	Length (cm)	Diameter (cm)
1	Ultra-high-permeability reservoir	Fine sandstone	30.96	3956	3.53	2.47
2	Ultra-high-permeability reservoir	Fine sandstone	30.26	2568	3.83	2.47
3	High-permeability reservoir	Siltstone	29.32	1890	4.94	2.49
4	High-permeability reservoir	Siltstone	28.84	1398	4.73	2.48
5	Medium-permeability reservoir	Siltstone	27.79	381	4.03	2.47
6	Medium-permeability reservoir	Siltstone	27.33	238	3.91	2.48

**Table 2:** Selection of rock samples at different sizes and simulated parameter settings.

Reservoir Type	Sample Number	Resolution Size (μm/voxel)	Dimension Size (μm)	Boundary Condition	Reynolds Number
Ultra-high-permeability and high-permeability reservoirs	24	2, 4, 8	800, 1200, 1600, 2000	Pressure Boundary	~0.01
Medium-permeability reservoir	5	1, 2, 4	440, 520, 580, 640		

**Table 3:** Classification criteria for local pore structure in reservoir rocks.

Pore Structure Type		Criteria for Classification	
		Pore Diameter/μm	Flux Area/μm^2^
Large pore	High connectivity	>84	>313
	Low connectivity	>84	≤313
Medium pore	High connectivity	(58, 84]	>313
	Low connectivity	(58, 84]	≤313
Small pore	High connectivity	≤58	>313
	Low connectivity	≤58	≤313

**Table 4:** Design of displacement-collaborative computed tomography (CT) scan experiment.

Sample Size	Diameter 4 mm, Length 8 mm, Scanning Resolution 2 μm							
Displacement Nodes	Dry Sample	Oil Injection	0.5 PV	1.0 PV	3.0 PV	10.0 PV	50.0 PV	500.0 PV
Displacement Speed (μm/s)	/	/	11	11	11	33	110	110

**Figure A2:**
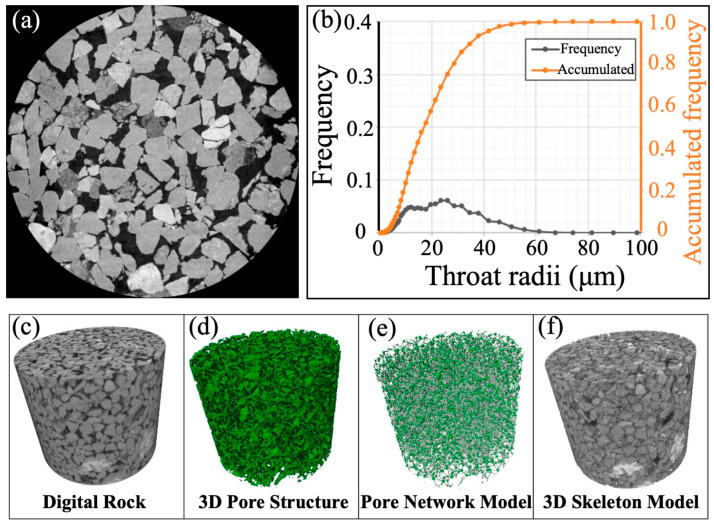
The micro-CT image data and postprocess: (**a**) gray images from CT scanning; (**b**) pore size radius distribution on digital rock; (**c**) digital rock; (**d**) 3D pore structure; (**e**) pore network model; and (**f**) 3D skeleton model.

## Data Availability

The pore network extraction in this work is based on the open-source software local resistance equivalence pore-throat segmentation (LoRePorTS V3). The library is available at Mendeley Data via https://data.mendeley.com/datasets/mwsxdb2tpm/3 (accessed on 25 October 2022).

## Abbreviations

SEM	Scanning electron microscopy
Micro-CT	Micro-computed tomography
FIB-SEM	Focused ion beam-scanning electron microscopy
NMR	Nuclear magnetic resonance
REV	Representative element volume
LBM	Lattice Boltzmann method
PV	Pore volume

## Appendix A. Micro-CT Imaging Setup and Data Analysis Description

### Appendix A.1. Micro-CT Imaging Equipment and Parameters

All micro-CT scans in this study were conducted using the Xradia MicroXCT-400 system, a high-resolution, non-destructive X-ray imaging device capable of multi-scale scanning, as shown in Figure A1. The system supports a large field-of-view (FOV) imaging mode while maintaining micron-scale spatial resolution, making it particularly suitable for studying complex pore structures in heterogeneous reservoir rocks.

- Model: X-radia MicroXCT-400;
- Nominal spatial resolution: ~3.5 μm per voxel;
- X-ray source: Tungsten anode with adjustable voltage (40–150 kV) and power (up to 10 W);
- Detector type: 4× sCMOS-based camera system;
- Sample size: Up to 40 mm in diameter;
- Field of View (FOV): 8–25 mm (depending on resolution setting);
- Scanning mode: 360° full rotation with 1000–1600 projections per sample.

Each core plug was scanned before and after high-PV waterflooding to monitor structural changes in the pore system. Imaging parameters were kept consistent across all scans to ensure comparability.

### Appendix A.2. Raw Image Examples and Data Processing Outputs

To support the analysis and improve transparency, we provide key examples of the imaging data and derived models used throughout this study.

(a) Raw micro-CT 2D Slice Images

Representative raw grayscale images (single slice) of the sandstone cores are shown in Figure A2a. These slices illustrate the internal heterogeneity of the matrix and pore phases, with visible contrast allowing for precise segmentation using thresholding-based image processing algorithms.

(b) Throat and Pore Size Distribution Analysis

Based on the grayscale images, we applied a watershed-based segmentation method to identify pore bodies and throats. The resulting pore-throat size distribution is illustrated in Figure A2b, showing the relative frequency of characteristic lengths in each sample, which serves as a quantitative foundation for classification.

(c) Reconstructed Digital Rock Models

Using filtered and segmented CT data, we reconstructed 3D digital rock models (Figure A2c,d), capturing pore morphology and connectivity. These models were used to compute porosity, connectivity, and flow pathways, and served as the input for further structural classification.

(d) 3D Pore Network and Skeleton Models

To further investigate topological features, we extracted pore network models (PNMs) and medial-axis skeletons from the segmented data. These are shown in Figure A2e,f, where nodes represent pores and links represent throats, providing structural input to evaluate pore-scale flow characteristics.

### Appendix A.3. Summary of Microstructural Quantification Workflow

**Table A1:** Summary of Microstructural Quantification Workflow.

Step	Description
1	Micro-CT scanning (pre- and post-flooding) using X-radia MicroXCT-400
2	Image filtering and grayscale normalization
3	Thresholding and segmentation of pore space
4	Morphological analysis to extract size distributions
5	3D reconstruction of digital rock
6	Network extraction and skeletonization for topological analysis
7	Pore classification based on size and local flux area

This workflow supports the pore classification framework introduced in the manuscript, providing a bridge between imaging data and flow-related structural interpretations.

## References

[1] Hou L.H., Li H.W., Li Q. (2018). Characteristics of oil and gas discoveries in recent 20 years and future exploration in the world. Pet. Explor. Dev..

[2] Zhang H., Shan G.J., Du Q.L., Wang C.X. (2022). Technical challenges and solutions of water flooding development in late stage of ultra-high water cut in placanticline oilfield in Daqing. Pet. Geol. Oilfield Dev. Daqing.

[3] Yuan Q.F., Pang Y.M., Du Q.L., Fang Y.J., Zhao Y.F., Lu H.M. (2017). Development laws of the sandstone oilfields at extra-high watercut stage. Pet. Geol. Oilfield Dev. Daqing.

[4] Liu K., Wang R., Shi W.Z., Martín J.D., Qi R., Zhang W., Qin S., Travé A. (2023). Full scale of pore-throat size distribution and its control on petrophysical properties of the Shanxi Formation tight sandstone reservoir in the North Ordos Basin, China. Lithosphere.

[5] Li Q., You X., Li J., Zhou Y., Lu H., Wu S., Yue D., Zhang H. (2024). Pore structure and factors controlling shale reservoir quality: Acase study of Chang 7 Formation in the Southern Ordos Basin, China. Enerigies.

[6] Keith W.S., Robert M.C. (2015). The evolution of pore-scale fluid-saturation in low-permeability sandstone reservoirs. AAPG Bull..

[7] Coskun S.B., Wardlaw N.C. (1996). Image analysis for estimating ultimate oil recovery efficiency by waterflooding for two sand-stone reservoirs. J. Petrol. Sci. Eng..

[8] Zhou Y., Wu S., Li Z., Zhu R., Xie S., Zhai X., Lei L. (2021). Investigation of microscopic pore structure and permeability prediction in sand-conglomerate reservoirs. J. Earth Sci..

[9] Xiao L., Bi L., Yi T., Lei Y., Wei Q. (2023). Pore structure characteristics and influencing factors of tight reservoirs controlled by different provenance systems: A case study of the Chang 7 Members in Heshui and Xin’anbian of the Ordos Basin. Energies.

[10] Mu C., Hua H., Wang X. (2023). Characterization of pore structure and reservoir properties of tight sandstone with CTS, SEM, and HPMI: A case study of the tight oil reservoir in fuyu oil layers of Sanzhao Sag, Songliao basin, NE China. Front. Energy Res..

[11] Dai Z., Luo D.L., Xie M.Y. (2019). A new method for particle size analysis of cuttings images based on improved watershed algorithm. China Offshore Oil Gas.

[12] Jiang Z., Pan Y., Fu C., Li W., Wang Y., Long W. (2023). Three-dimensional pore structure characterization of cement paste by X-ray computed tomography (XCT) and focused ion beam/scanning electron microscopy (FIB/SEM). Constr. Build. Mater..

[13] Bijoyendra B., Sushanta K., Douglas V. (2011). Understanding the micro structure of Berea Sandstone by the simultaneous use of micro-computed tomography (micro-CT) and focused ion beam-scanning electron microscopy (FIB-SEM). Micron.

[14] Gu N., Hu W., Gao L., Liu G. (2024). Three-dimensional spatial microscopic characteristics and developmental influencing factors of tight gas layers in Hangjinqi Prospect Area, Ordos Basin, China. Energies.

[15] Sakdinawat A., Attwood D. (2010). Nanoscale X-ray Imaging. Nat. Photonics.

[16] Ju Y., Gong W., Xie H., Chang C., Xie L., Liu P. (2017). Three-dimensional characterisation of multi-scale structures of the Silurian Longmaxi shale using focused ion beam-scanning electron microscopy and reconstruction technology. J. Nat Gas Sci. Eng..

[17] Dmitriy A.M., Shadfar D., Ali K., Masoud R., Yousef K., Ma T.S. (2024). Multiscale and diverse spatial heterogeneity analysis of void structures in reef carbonate reservoirs. Geoenergy Sci. Eng..

[18] Ali A.A., Namam M.S., Dmirriy A.M. (2023). Paleoenvironmental evaluation using an integrated microfacies evidence and triangle model diagram: A case study from Khurmala Formation, Northeastern Iraq. J. Mar. Sci. Eng..

[19] Ju Y., Xi C., Zheng J., Gong W., Wu J., Wang S., Mao L. (2022). Study on three-dimensional immiscible water–Oil two-phase dis-placement and trapping in deformed pore structures subjected to varying geostress via in situ computed tomography scanning and additively printed models. Int. J. Eng. Sci..

[20] Zhang J., Sun J., Cao Y., Zhong X., Li S. (2023). Research of micro-pore structure and seepage characteristics of Portugal Reservoir in Lamadian Oilfield. Miner. Explor..

[21] Wang J., Wu S., Xiao S., Guo S., Lv Z., Jiao H., Guo Z., Liu Z., Wu H., Xiao Q. (2021). Distribution characteristics of micropore throat size of turbidite sandstone reservoir in middle sub-member of 3rd member, Shahejie Formation in Dongying Depression. J. China Univ. Pet..

[22] Lin J., Yang Y., Yin J., Liu Y., Li X. (2023). Study on pore structure evolution characteristics of weakly cemented sandstone under freeze–thaw based on NMR. Water.

[23] Chen D., Zhu Y., Wang W., Zhang L., Tang J., Ren J., Wang Y. (2024). Pore structure of tight sandstones with differing permeability: The He 8 Member of the Middle Permian Lower Shihezi Formation, Gaoqiao area, Ordos Basin. Energy Sci. Eng..

[24] Zhang Q., Qi H., Huo Y., Li Y., Li T., Zhang D., Lin K., Yang C., Tong J., Zhao H. (2023). Study of pore-throat structure characteristics and fluid mobility of Chang 7 tight sandstone reservoir in Jiyuan area, Ordos Basin. Open Geosci..

[25] Chen C., Fu L., Chen X., Zhang T., Xie Y., Wang H., Zhu Y. (2021). Quantitative Evaluation Method for Micro Heterogeneity of Tight Sandstone: A case study of Chang-6 reservoir of Yanchang Formation in Huaqing area, Ordos Basin. Acta Sedimentol. Sin..

[26] Gong W., Liu Y., Xi C., Ju Y., Wang M. (2024). Dynamic characterization of residual oil during long-term waterflooding experiments in heterogeneous porous structures. Fuel.

[27] Ren X., Li A., Wang Y., Wu S., Wang G. (2015). Pore structure of tight sand reservoir and its influence on percolation-taking the Chang 8 reservoir in Maling oilfield in Ordos basin as an example. Oil Gas Geol..

[28] Guo H., Liu Q., Li H., Meng Z., Bai Y. (2013). Characteristics of pore structure of Jurassic tight reservoir in Sichuan basin. J. Shenzhen Univ..

[29] Pei Z., Zhou Z., Chang W., Zhang Y. (2022). Type division and reservoir characteristics of micro-pore structure in S area of Daqing placantieline. Pet. Geol. Eng..

[30] Lin W., Li X., Yang Z., Xiong S., Luo Y., Zhao X. (2020). Modeling of 3D rock porous media by combining X-Ray CT and Markov chain Monte Carlo. J. Energy Resour. Technol.-Trans. ASME.

[31] Yun L. (2020). Reconstruction and analysis of tight sandstone digital rock combined with X-ray CT scanning and multiple-point geostatistics algorithm. Math. Probl. Eng..

[32] Song R., Wang Y., Liu J., Cui M., Lei Y. (2019). Comparative analysis on pore-scale permeability prediction on micro-CT im-ages of rock using numerical and empirical approaches. Energy Sci. Eng..

[33] Fatt I. (1956). The network model of porous media. SPE.

[34] Liu Y., Gong W., Zhao Y., Jin X., Wang M. (2022). A Pore-Throat Segmentation Method Based on Local Hydraulic Resistance Equivalence for Pore-Network Modelling. Water Resour. Res..

[35] Meng J., Zhang L.Y., Li R., Zhao A.F., Zhu B.W., Huang P., Shen S.B. (2023). Microscopic pore structure characteristics of tight sandstone reservoirs and its classification evaluation. Spec. Oil Gas Reserv..

[36] Pang Y., Liu Y., Zhang L., Xi N., Yuan L., Chen C. (2023). Micro-pore structure and fluid mobility of tight sandstone reservoirs of Chang 8 menber in Huachi area in Ordos basin. Pet. Geol. Oilfield Dev. Daqing.

[37] Ran X., Wu S., Fu J., Wei X., Chu M. (2013). Research on the pore structure classification of low permeability reservoir of the Yanchang formation in Longdong area, Ordos basin. Earth Sci. Front..

[38] Yang Y., Li Y., Yao J., Zhang K., Iglauer S., Luquot L., Wang Z. (2019). Formation damage evaluation of a sandstone reservoir via pore-scale X-ray computed tomography analysis. J. Pet. Sci..

[39] Wang F., Wang X., Liu Y., Deng Q., Xu J., Zhang Y., Li H. (2021). The Evolutionary Characteristics of Reservoir Microstructure under Long-Term Waterflooding Development and Its Fractal Description. Geofluids.

[40] Zhou Y., Yang W., Yin D. (2022). Experimental investigation on reservoir damage caused by clay minerals after water injection in low permeability sandstone reservoirs. J. Pet. Explor. Prod. Technol..

